# Supplementary material for: Acontia, a Specialised Defensive Structure, Has Low Venom Complexity in Calliactis polypus
Source: Toxins (Basel). 2023 Mar 12;15(3):218. doi: 10.3390/toxins15030218 (PMC10051995; doi:10.3390/toxins15030218)
Supplement: Supplementary file 1 [file toxins-15-00218-s001.zip › toxins-2260107-Supplementary Material S1.pdf]

Supplementary Materials: Acontia, a Specialised Defensive Structure, Has Low Venom Complexity in *Calliactis polypus*

Hayden L. Smith, Peter J. Prentis, Scott E. Bryan, Raymond S. Norton and Daniel A. Broszczak

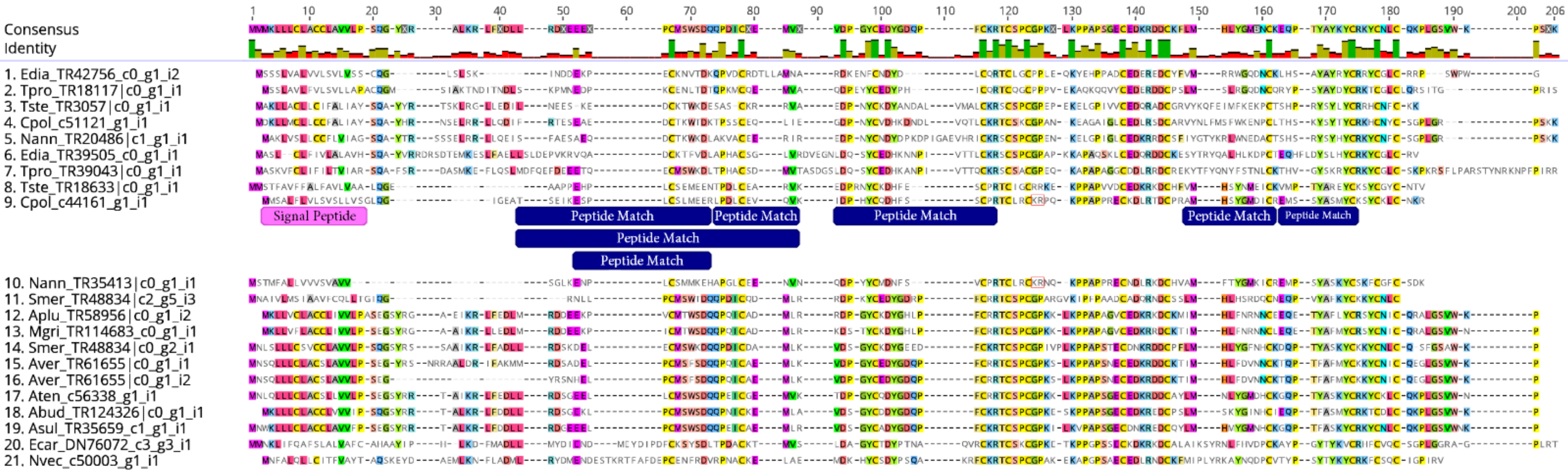

Figure S1. Alignment of amino acid sequences for the Unknown 12C candidate genes and the consensus sequence and identity percentage above. Annotation of the candidate identified in the *Calliactis polypus* acontia proteome is depicted with the signal peptide and peptide matches from the mass spectra of the SWATH-MS and DDA pooled acontia sample. Consensus identity percentages are as follows, green bar for 100 %, yellow bar for ≥ 50 % and < 100 %, and red bar for < 50 %.
